# A spatial examination of alcohol availability and the level of disadvantage of schools in Ireland

**DOI:** 10.1186/s12889-024-18261-y

**Published:** 2024-03-13

**Authors:** Anne Doyle, Ronan Foley, Frank Houghton

**Affiliations:** 1 Health Research Board, Grattan House 67–72 Lower Mount Street, Dublin, Ireland; 2 Maynooth University, Maynooth, Co. Kildare Ireland; 3 Technological University of the Shannon, Limerick, Ireland

**Keywords:** Alcohol, Liquor licencing, Disadvantaged schools

## Abstract

**Background:**

The availability of alcohol is a major factor in underage drinking and according to the alcohol harm paradox, those living in more deprived communities are more susceptible to the negative consequences of alcohol use, despite drinking the same or less than those from more affluent areas. Alcohol availability within the vicinity of the home or school normalises alcohol for schoolchildren. For the first time in the Republic of Ireland, this study examines the number of premises licensed to sell alcohol within 300 m of all schools in Ireland and differences in this number between disadvantaged and non-disadvantaged schools.

**Methods:**

Using publicly available data from the Department of Education and Revenue, the addresses of all schools (*n* = 3,958) and all premises with at least one liquor licence (*n* = 14,840) were geocoded and analysed using the Geographic Information System software, Quantum GIS (QGIS). Schools were identified by their disadvantaged classification using the HP Pobal Deprivation Index and the number of liquor licences within 300 m of each school type was examined. To test for significant differences between schools’ level of disadvantage, a combination of Mann-Whitney U tests, Kruskal-Wallis tests and Dunn-Bonferroni tests were used.

**Results:**

There was a mean of two licenced premises within 300 m of all schools in Ireland, but when disadvantaged schools were compared to non-disadvantaged schools, there was a significantly higher number of licenced premises around disadvantaged schools (*p* < .001). Primary schools are further classified according to their level of disadvantage and the results indicated that those schools classified as the most disadvantaged had a significantly greater number of liquor licences within 300 meters (*p* < .001). There was no significant difference in density of licenced premises when comparing disadvantaged secondary schools with non-disadvantaged secondary schools (*p* = .705).

**Conclusion:**

Ireland is considering increasing alcohol availability through the Sale of Alcohol Bill, 2022. However, this analysis indicates already problematic numbers of licenced premises within close proximity of schools in Ireland. It is essential that the harms associated with alcohol availability are considered, especially for those living and attending school in disadvantaged communities, where higher numbers of licenced premises were identified.

## Introduction

Alcohol is a significant commercial determinant of health as its use can have detrimental effects on young people’s lives. Globally, for those aged 10–24-years, alcohol use was the second leading risk factor attributable to deaths and disability-adjusted life years (DALYs) in 2019 [1]. Alcohol contributes to significant health and social harms for all age groups, and children and adolescents are especially vulnerable as they are developing physically, psychologically and emotionally [2]. During adolescence, the brain is developing at a rapid pace and continually exposed to an abundance of information by the people and environment around them. Decision-making is strongly influenced by social and environmental factors, and although familial factors also contribute to influencing children to initiate alcohol use, marketing and availability of alcohol are key drivers of youth drinking [3–7]. Survey data reveal that schoolchildren in Ireland report that buying alcohol in the locality where they live and go to school is ‘easy’, and the evidence available indicates that alcohol is widely promoted in our society [8, 9].

Per capita alcohol use among the entire population in Ireland has decreased since a peak in 2001 (14.3 L). However, it remains high (10.2 L in 2022), higher than the Department of Health target of 9.1 L target for 2020 [10, 11]. Alcohol is deeply embedded in all aspects of our lives, not only in social and celebratory events, but also in daily living. In such an alcogenic environment, it is unsurprising that alcohol use among adolescents is highly prevalent in Ireland [2]. According to the Irish Health Behaviour in School-aged Children (HBSC) survey, 50% of 15-year-olds, 70% of 16-year-olds, and 82% of 17-year olds have consumed alcohol in their lifetime [8]. Heavy episodic drinking (HED) is also a prevalent practice among adolescents; one-third of 15–16 year-olds in Ireland reported last month HED in 2019 (33%) according to the European School Survey Project on Alcohol and Other Drugs (ESPAD) [12]. Rates of HED in the last year among female adolescents in Ireland are ranked at 3rd highest (and male adolescents at 4th highest) in a Lancet study examining 195 countries between 1990 and 2016 [13].

Goal 1 of the national drugs strategy in Ireland Reducing Harm, Supporting Recovery: A health-led approach to drug and alcohol use in Ireland 2017–2025, aims to prevent early alcohol and other drug use among young people [14]. Children and adolescents are also central to the Public Health (Alcohol) Act, 2018, Irish legislation that acknowledges our high per capita alcohol use and related harms [15]. The objectives of the Act are to reduce alcohol use and harm at a population level but specifically to prevent and delay children from alcohol use and the underlying principles of the Act are based on the World Health Organization’s ‘best buys’ (policies that are cost-effective, evidence-based, and yield a significant return on investment for governments to adopt) to reduce alcohol–related harms [16]. In an effort to achieve its goal of delaying and preventing children’s alcohol use, Sect. 14 of the Public Health (Alcohol) Act, 2018 prohibits alcohol advertising in parks and public open spaces, on public transport (vehicles and stations), and within 200 meters of the perimeter of schools, playgrounds and child services locations [15]. However, the legislation does not apply to premises selling alcohol (e.g. shops and pubs etc.) and 44% of all schools (both primary and secondary) in Ireland have at least one licenced premises within 300 meters of the school [17].

Alcohol outlets in the vicinity of schools normalise alcohol and acceptance of alcohol use and children are regularly exposed to this form of alcohol advertising [17–20]. High alcohol outlet density is associated with increased price competition, making alcohol more affordable and accessible, two of the main driving forces for adolescence alcohol use [18, 21]. The available evidence suggests that greater exposure to alcohol advertising influences adolescents to initiate alcohol use and engage in HED and hazardous drinking [22, 23]. The evidence also indicates that greater alcohol availability and density of liquor licences within a community is associated with higher levels of alcohol use among that population [24–26]. This also applies when premises selling alcohol are in close proximity to schools or where schools are situated in areas densely populated with liquor licences. For example, closer proximity and higher density of liquor licences is associated with early alcohol initiation and higher rates of adolescent drinking [6, 7, 26–33]; truancy, lower academic achievement, and disruptive behaviour in class [34, 35]; and violence, crime and domestic violence [30, 36–38]. Neighbourhoods in deprived areas are more likely to have a higher density of premises selling alcohol, a cause-effect of the alcohol harm paradox (AHP), where those in deprived communities are more likely to experience alcohol-related harms despite drinking the same or less than those in more affluent neighbourhoods [30, 39–41]. This applies to schools too as those in more deprived areas are more likely to have a higher number of alcohol outlets [20, 28, 32]. However, alcohol availability, specifically comparing areas of deprivation, has not been considered in Ireland to date. One exception is a recent commentary that examined the number of liquor licences around all schools in Ireland but did not factor disadvantage [17].

The Sale of Alcohol Bill, 2022, aims to stimulate economic activity in Ireland, particularly in the larger cities, and proposes to increase the number of premises permitted to sell alcohol by removing the extinguishment requirement [42]. Currently the law stipulates that if an individual or business wishes to open a licenced premises, they cannot do so without first acquiring an existing licence, for example, a small, rural pub can sell their licence to a large supermarket chain to be used for the purposes of an in-store off-licence in an urban area. Without the extinguishment requirement, there would be no limit on the number of alcohol licenses as any ‘cultural amenity’ can apply for a licence to sell alcohol without extinguishing an existing licence.

The Bill also proposes to extend opening hours of licenced premises. Concern has been widely raised about the potential negative consequences of this [38, 43]. Considering that adolescents in Ireland are among the highest globally for levels of binge drinking and considering the inequalities driven by the AHP, this study sought to determine: what is the density and proximity of liquor licences in relation to schools in Ireland? Does this differ depending on the level of disadvantage of the school (based on Delivering Equality of Opportunity In Schools (DEIS) status versus non-DEIS school status)? Alcohol availability in areas of deprivation has been examined in other countries but not to date in the Republic of Ireland. This analysis will fill a gap in the research literature in Ireland as it is the first of its kind to study alcohol availability surrounding disadvantaged schools. The findings can be used as part of a health impact assessment in response to proposed new legislation in Ireland and contribute to policies addressing density of alcohol availability especially in deprived communities.

## Methodology

According to the Department of Education, there were 3,958 mainstream schools in Ireland for the school year 2022/2023 (3,231 primary schools and 727 secondary schools) [44]. Primary schools (also referred to as national schools) had 549,198 children aged approximately 5 years to 12 years enrolled for the school year 2022/2023 and secondary schools (post-primary) had 193,773 adolescents aged approximately 13 years to 18 years enrolled for the school year 2022/2023. Of these, 31.2% of primary schools (*n* = 966) and 32.3% of secondary schools (*n* = 235) were classed as DEIS schools for the school year 2022/2023.

A school is classified as a DEIS school according to the number of children enrolled who are from disadvantaged backgrounds defined by the level of income within the household and using the HP Pobal Deprivation Index derived from census data to measure the relative affluence or disadvantage of small areas [45]. The Education Act, 1998 recognises that some children from disadvantaged backgrounds may experience educational disadvantage compared to their peers and this can result in poor levels of participation in the educational system and lower levels of achievement [46]. In response to this, in 2005, the Department of Education published their action plan to address how the education system in Ireland needed to adapt a more integrated approach to educational inclusion, and a standardised system for identifying levels of disadvantage in the form of DEIS was adopted [47]. The DEIS Plan in 2017 included further actions to develop best practice in the identification of schools needing support including enhancing the learning experience and academic achievements of pupils in DEIS schools, piloting innovative and creative approaches to tackle educational inequality, and providing DEIS schools with the necessary research, information, evaluation and feedback to monitor in achieving the plan’s objectives. Schools classified as DEIS receive additional funding from the Department of Education and in 2022, the DEIS identification model was refined and resulted in an expansion of the number of schools classified as DEIS to target resources to schools with the highest concentration of students at risk of educational disadvantage. This included introducing ‘bands’ in the classification of disadvantage, and primary schools are now categorised according to the most concentrated levels of disadvantage [48]. Urban primary schools facing the highest levels of concentrated disadvantage are classified as Urban Band 1, receiving the greatest amount of support and resources under the DEIS plan, reflecting their significant needs. Urban band 1 schools are more likely to be situated in cities and large towns in Ireland. Urban Band 2 comprises primary schools in urban areas with high levels of concentrated disadvantage and Rural DEIS comprises primary schools in rural areas experiencing high levels of concentrated disadvantage. Secondary schools have just one classification of deprivation and all DEIS secondary schools receive an equal amount of additional funding regardless of their location.

### Spatial data collation

In the school year 2022/2023, there were 153,709 pupils enrolled in DEIS primary schools and 103,646 pupils enrolled in DEIS secondary schools, representing 25.6% of primary school pupils and 28.0% of secondary school pupils, representing 26.9% of pupils overall, or over 1 in 4 pupils benefiting from the programme. The publicly available details of the school include full address including Eircode (i.e., postal or ZIP code), DEIS status, the number of pupils and XY coordinates of the school. This point address formed the basis of subsequent 300-meter buffer analysis conducted using QuantumGIS (QGIS). Roughly speaking a typical person walks the following distances in one minute: 90 m at a brisk pace; 60–70 m at a moderate speed; 40–50 m at a slow pace; and 30–40 m at a very slow pace [49]. Eircodes were then geocoded using Geographic Information System (GIS) software, QuantumGIS (QGIS). This analysis focussed on the disadvantage level of schools examined. Local area-based deprivation was not examined given the high number of children that commute relatively long distances to school in Ireland, particularly in rural areas and for well-performing schools [50].

A list of liquor licences issued or renewed for premises for the period 2022–2023 (*n* = 14,840) was obtained from Revenue [51]. This list is updated annually and includes the name and address of the licensee, the address of the premises and the type of liquor licence held. Revenue issues various different licences in Ireland including, publican licences which includes pubs, hotel bars, theatres, holiday camps, greyhound racetracks, horse racetracks, and railway refreshment rooms; off-licences permitting the sale of alcohol for consumption off the premises; wine retailers on-licence permitting the sale of wine, sherry, and fermented liquor containing less than 23% alcohol by volume (ABV); special restaurant licences permitting alcohol sales for consumption on the premises to persons who have ordered a meal; wholesale dealer’s licence allowing the sale of alcohol in bulk quantities; manufacturer’s licence allowing the manufacturing of liquor and the wholesale of the manufactured liquor from the manufacturing premises [51]. Address data can be geocoded to point locations using a Geographic Information System. Unfortunately, 20% (*n* = 2,685) of the addresses from the Revenue list were incomplete. To overcome this, manual geocoding was performed for each address with insufficient information using Google Maps [52].

### Statistical analysis

Addresses of the licenced premises were then geocoded and imported into QGIS. In order to examine the location of liquor licences and their proximity measures (300 m) and density to school disadvantage level (DEIS vs. non-DEIS), a specific ‘database join’ command was applied using QGIS. The 300-meter buffer zone from the point location of the school was used as this distance has been considered in previous studies, for example, Martín-Turrero et al., who used sensitivity analysis for different buffer sizes, and because of the legislation in Ireland restricting alcohol advertising within 200 m proximity of school perimeters [15, 28, 53].

Data to examine differences in the number of liquor licenced premises surrounding schools by disadvantaged status were analysed using IBM SPSS Statistics (Version 29). Mann-Whitney U tests, Kruskal-Wallis tests and Dunn-Bonferroni tests were conducted to specifically examine significance between DEIS and non-DEIS, levels of DEIS, and primary compared to secondary schools.

## Results

Preliminary analysis examined all primary and secondary schools combined (*n* = 3,822). The mean number of licensed premises within 300 m of all Irish schools was 2.01 (SD = 4.8, median = 0, IQR = 0–2). The mean number of licensed premises within 300 m of non-DEIS schools was 1.75 (SD = 4.43, median = 0, IQR = 0–2) and for all DEIS schools, it was 2.61 (SD = 5.58, median = 1, IQR = 0–2). A Mann-Whitney U test was conducted to explore differences in the number of licenced premises within 300 m of all schools on the basis of DEIS categorisation. The results indicated that DEIS schools had significantly higher numbers of licensed premises nearby than non-DEIS schools, z = -6.696 (1201) (2757), *p* < .001.

### Primary schools and liquor licence density

**Fig. 1:**
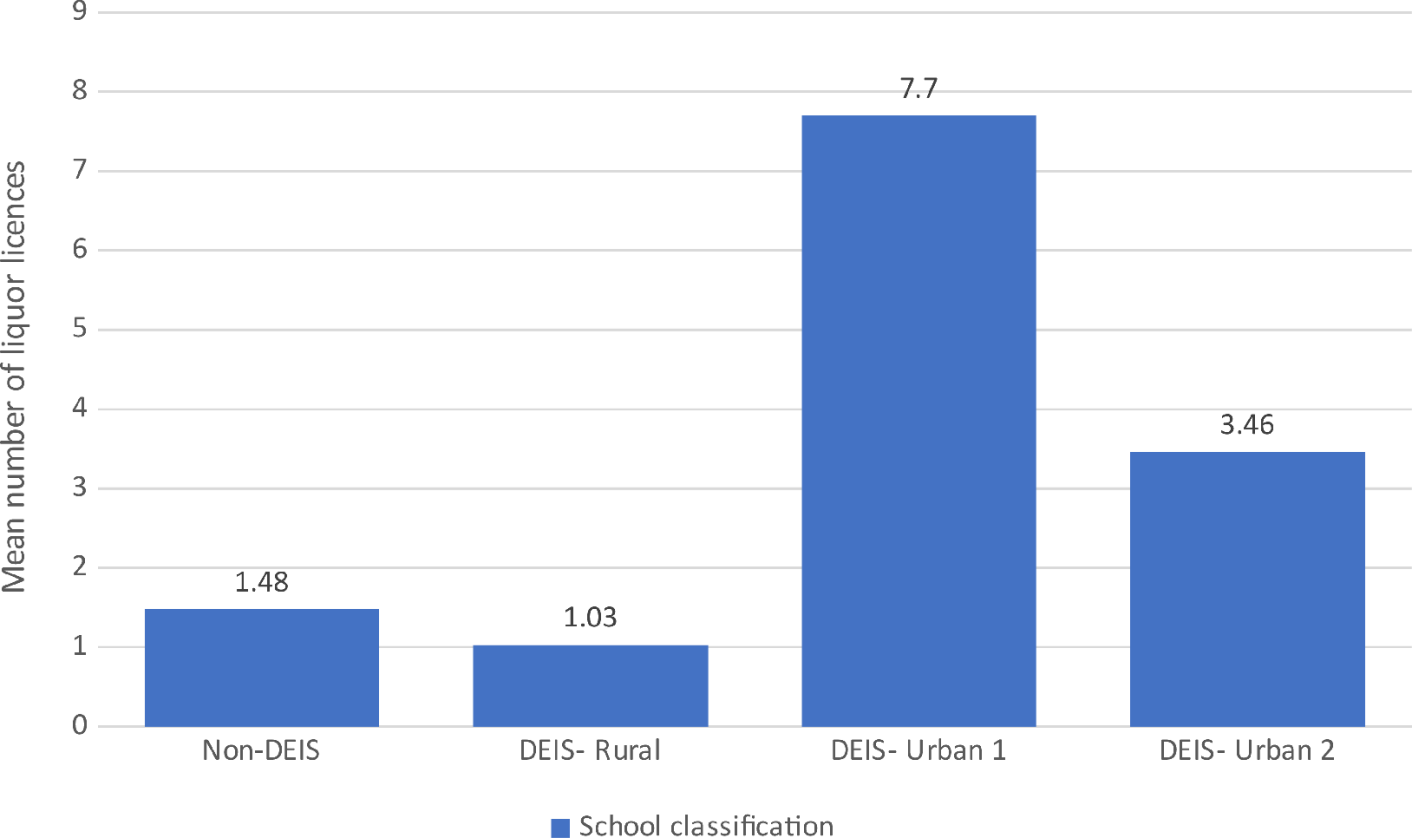
Mean average number of liquor licences within 300 m of primary schools by DEIS classification

Figure 1 illustrates the mean number of premises licenced to sell alcohol within 300 m of primary schools in Ireland broken down DEIS category.

The mean number of liquor licenses within 300 m of primary schools designated as in the DEIS Urban 1 category is particularly problematic at 7.7 (Table 1). As the data is skewed, medians and the inter-quartile range (IQR) is given alongside means and standard deviations (SD).

**Table 1:** Number of liquor licences within 300 m of primary schools by DEIS categorisation

	All primary schools	Non-DEIS primary schools	DEIS primary schools	DEIS Rural primary schools	DEIS Urban 1 primary schools	DEIS Urban 2 primary schools
N	3231	2265	966	509	306	151
Mean	1.79	1.48	2.53	1.03	7.70	3.46
Std Dev	4.48	3.96	5.44	2.27	4.58	5.94
Median	0.00	0.00	1.00	0.00	1.00	1.00
IQR	0–2	0–1	0–2	0–1	0–5	0–4
Range	0–73	0–73	0–41	0–19	0–41	0–34

A Kruskal-Wallis test indicated that there was a significant difference in the number of liquor licenses within 300 m of primary schools across DEIS categories, χ2 ([df = 3], *N* = 3231) = 185.224, *p* < .001. The median number of liquor licences within 300 m of primary schools were as follows: 0 for DEIS - Rural schools, 1 for DEIS Urban 1 schools, 1 for DEIS Urban 2 schools, and 0 for non-DEIS schools.

Post-hoc comparisons using Dunn’s method with a Bonferroni correction for multiple tests indicated that the median number of liquor licences within 300 m of non-DEIS primary schools was significantly lower than that of DEIS Urban 1 primary schools, *p* < .001 and DEIS Urban 2 primary schools *p* < .001. This analysis also indicated that the median number of liquor licences within 300 m of DEIS rural primary schools was significantly lower than that of DEIS Urban 1 primary schools, *p* < .001 and DEIS Urban 2 primary schools *p* < .001. However, there was no significant difference between the median number of liquor licences within 300 m of non-DEIS primary schools and DEIS primary schools, *p* = .820. Similarly, there was no significant difference between the median number of liquor licences within 300 m of DEIS Urban 1 primary schools and DEIS Urban 2 primary schools, *p* = .285.

### Secondary schools and liquor licence density

Table 2 examines the results for secondary schools. Although the results are broadly similar between non-DEIS and DEIS schools, they are also problematic with a mean average of almost 3 premises licenced to sell alcohol within 300 m of all secondary schools in Ireland.

Non-parametric analysis (Mann-Whitney U) found that there was no significant difference between the number of liquor licences held within 300 m of DEIS versus non-DEIS secondary schools, z = -0.378 (235) (492), *p* = .705.

**Table 2:** Number of liquor licences within 300 m of secondary schools by DEIS categorisation

	All Secondary Schools	Non-DEIS Secondary Schools	DEIS Secondary Schools
N	727	492	235
Mean	2.95	2.97	2.92
Std Dev	6.02	6.00	6.14
Median	1.00	1.00	1.00
IQR	0–3	0–3	0–2
Range	0–50	0–45	0–50

## Discussion

This is the first time an analysis of alcohol availability around disadvantaged schools in Ireland has been conducted. Using a complete list of all schools in Ireland for the academic year 2002/2023 and addresses of all premises issued with a licence to sell alcohol for the period 2022–2023, the density of liquor licences within 300 m of schools in Ireland were mapped using a GIS. This study found a high number of premises licenced to sell alcohol within close vicinity of both primary and secondary schools, with an average of 2 licenced premises within just 300 m of every school in Ireland.

Schools in Ireland receive additional funding when they are located in an area of disadvantage and where a proportion of pupils are from disadvantaged backgrounds. This study compared the density of licences in close proximity to disadvantaged schools and non-disadvantaged schools and found that disadvantaged schools were significantly more likely to have a higher number of licenced premises within 300 m.

Primary schools in Ireland have further classification tiers according to their location and level of disadvantage and when examining primary schools separately, it was noted that primary schools classified as being the most disadvantaged (DEIS Urban 1) were significantly more likely to have a higher number of licenced premises within 300 m of the school compared to non-DEIS schools.

Furthermore, examination of secondary schools found no significant difference in the number of alcohol outlets in their proximity depending on their disadvantage status. However, the majority of adolescent pupils in the senior cycle have approximately 3 licenced premises within a short walking distance from their school.

The number of licenced premises observed around Irish schools is less than that obtained in a similar study examining their proximity to secondary schools in Madrid, Spain, which reported an average of 26 licenced premises [28]. However, that study was based in a major tourist city with a population of over 3 million and used a wider buffer zone (400 m) than the current study but did find a higher prevalence in areas of greater disadvantage. A further study in New Brunswick, Canada reported an average of between 3 and 16 licenced premises within 499 m of schools, more prevalent in low income neighbourhoods [32]. Elsewhere, 15.5% of schools in Perth, Australia were found to be within 800 m of a licenced premises [33]. Our study findings that children going to school in deprived areas are more exposed to, the sometimes subtle, advertising of alcohol on licenced premises in the vicinity of their school. Similarly in Glasgow, Scotland, areas of deprivation were more likely to have a greater density of alcohol outlets (as well as fast food, tobacco, and gambling outlets) [54]. Elsewhere, using GPS data to examine schoolchildren’s exposure to alcohol outlets, those living in more deprived communities were significantly more likely than their counterparts on less deprived areas, to be exposed to alcohol outlets, and much of this exposure was within 500 m of their home [20].

International evidence has shown that the presence of licensed premises near children’s schools and homes is linked to an increased likelihood of alcohol use, binge drinking, and detrimental effects on school behaviour [6, 26, 34, 35]. The finding from this study that the most disadvantaged primary schools in Ireland have the highest concentration of licensed premises within close vicinity is particularly concerning considering the alcohol harm paradox.

These findings emphasise the importance of addressing societal inequalities and their impact on health and alcohol-related behaviours. Deprivation significantly contributes to alcohol-related harm, highlighting the need for public health interventions to address these disparities. The Sale of Alcohol Bill, 2022, proposes to increase alcohol availability in Ireland despite the widespread existing evidence of potential harm this can result in. It is crucial that the evidence presented here be considered, along with the supporting international literature of the consequences of alcohol availability, especially near schools, and especially in areas of higher deprivation, and prioritise reducing this accessibility to reduce the adverse effects on schoolchildren and the wider community.

Inequalities in Irish society are prevalent in the Irish Government’s objectives and strategies, including the Department of Health Sláintecare strategy which aims to reduce inequalities; as well as the Irish National Drugs Strategy, where ‘communities’ and ‘deprivation’ are terms explicitly referred to as areas of priority [14, 55].

The Public Health (Alcohol) Act, 2018, aims to reduce per capita alcohol use and delay alcohol initiation among schoolchildren. The Act is based on best-practices recommended by the WHO, and includes a ban on alcohol advertising around schools. As licenced premises signage are exempt from this ban, it is important that planning of additional licenced premises is carefully considered, especially in areas of disadvantage. Alcohol use among schoolchildren in Ireland is high and rates of binge drinking among this cohort are among the highest in Europe. As those from disadvantaged backgrounds are more likely to experience alcohol-related harm despite similar alcohol use to peers from affluent backgrounds, careful planning of the issuing of additional licenses should be thought through. Communities can object to an application for a new licence or the renewal of a licence on the grounds of the character, misconduct or unfitness of the applicant, the unfitness or inconvenience of the new premises, unsuitability for the needs of persons residing in the neighbourhood, the adequacy of the existing number of licensed premises of the same character in the neighbourhood, the number of previously licensed houses in the neighbourhood, the manner in which a premises has been conducted in the previous year (e.g. convictions for ‘after hours’ trading, permitting drunkenness on the premises, serving to drunken or underage persons, etc.) and this will be especially important if the proposed legislation is enacted [56]. The findings from this study suggest greater regulation of licenced premises, particularly around schools and especially around schools in areas of greater deprivation could potentially reduce the proportion of adolescents engaging in underage drinking.

### Strengths and limitations

This study is the first, to the best of our knowledge, to examine liquor licence density surrounding all disadvantaged schools in the Republic of Ireland. The comprehensive lists of schools and licenced premises ensure that the data represent the whole of Ireland. However, this study has limitations. Firstly, the type of liquor licence issued to the premises was not considered (i.e. off-licence, publican etc.). Although all licenced premises types were included in the analysis (pubs, supermarkets, corner shops, standalone off licences etc.), future studies should consider the differences in proximity of the type of licence. Although supermarkets and smaller shops are essential to communities, the sale of alcohol is not, or the advertising of same and smaller shops are commonly frequented by schoolchildren thus increasing their exposure to alcohol and alcohol marketing.

Furthermore, schools categorised as disadvantaged, particularly primary schools classified as DEIS Urban Band 1 are more likely to be located in areas with higher population density where the availability of all types of retailers is high.

This study also did not examine the prevalence of alcohol use among the schoolchildren attending the schools, but the available evidence indicates a high percentage of schoolchildren in Ireland consume alcohol. It should be noted that the GIS analysis established the Euclidean distances of the licenced premises from schools (i.e., ‘as the crow flies’), further analysis may be useful to establish the distance and density using road (and pedestrian/path) network measurements. Furthermore, as Eircodes provide the exact x y coordinates of the main school building, those schools with multiple buildings or classrooms, or those with larger grounds, have a greater area and therefore the 300-meter buffer will differ depending on the geographic area of the school. Likewise, schools in built-up areas of towns and cities are more likely to have a higher density of outlets.

A further limitation is that this study did not examine the presence or the extent, of alcohol advertising on or surrounding the licenced premises. As 20% of the licenced premises were manually geocoded due to a lack of information, these addresses may be less accurate by using Google Maps compared to those geocoded using the Eircode.

## Conclusion

This study provides a valuable insight into the routine exposure schoolchildren in Ireland to alcohol and how accessible and available alcohol is within our communities, especially in deprived areas. This normalises alcohol use and the evidence indicates that this can have a potential harmful impact on children’s behaviour, impacting not only alcohol use among this cohort, but also affecting their performance and experience at school. As per other studies, our data indicate that there is a high number of licenced premises around schools in Ireland. Taking into account the known harms associated with this, it is important that proposed legislation to increase alcohol availability consider the health and societal impact of such action.

## Data Availability

A list of liquor licences issued or renewed is available publicly on the Revenue website: https://www.revenue.ie/en/corporate/information-about-revenue/statistics/excise/licences/liquor-licences.aspxA list of all schools in Ireland and their classification of disadvantage is available publicly from the Department of Education website: https://www.gov.ie/en/collection/63363b-data-on-individual-schools/.
